# Cancer nurses’ experience during the COVID-19 pandemic: Multicenter mixed-methods study on coping and resilience strategies

**DOI:** 10.1186/s12912-024-02085-7

**Published:** 2024-07-23

**Authors:** Lucia Cadorin, Cristina Mazzega-Fabbro, Sonja Cedrone

**Affiliations:** 1 Centro di Riferimento Oncologico di Aviano (CRO) IRCCS, Via F. Gallini, 2, Aviano, 33081 Italy; 2 University of Udine, Viale Ungheria, 49, Udine, 33100 Italy

**Keywords:** COVID-19, Pandemic, Coping strategies, Resilience, Mixed-methods, Oncology setting

## Abstract

**Background:**

In early 2020, the COVID-19 pandemic created severe difficulties in clinical and organizational fields. Healthcare workers needed to protect their health and avoid infecting their family members, but also limit the virus’s spread among vulnerable oncology patients undergoing hospital treatment.

**Objective:**

To evaluate the resilience and coping strategies of nurses working in the oncology setting.

**Methods:**

A mixed-methods study was conducted. First, two questionnaires (CD-RISK and COPE- NVI-25) were used to assess nurses’ resilience strategies and coping mechanisms quantitatively. Second, qualitative semi-structured interviews were conducted to explore the personal experiences of nurses who cared for patients during the pandemic, and Colaizzi’s framework was used for content analysis.

**Results:**

The 164 participants, the majority of whom were women (88.4%), reported high resilience. The CD-RISK score varied according to education. With respect to COPE-NIV-25, transcendent orientation and avoidance strategies had the lowest mean scores, while problem orientation was higher in nurses aged ≥ 40. Five themes emerged: (1) changes in work and personal areas; (2) feelings/emotions, such as fear of infection of themselves or their loved ones, difficulty in using the face mask, relational repercussions with patients or their families; (3) personal and working group strategies used to counteract the suffering attributable to COVID-19; (4) professionalism/nursing responsibilities in developing new rules and protocols, and (5) metaphors to describe their experiences.

**Conclusions:**

The COVID-19 pandemic led to major changes in the nurses’ roles, but they showed resilience and generated a positive working climate.

**Implication for practice:**

Even in emergency situations, nursing administrations and policymakers ought to ensure that nurses receive adequate training and support to develop resilience and coping strategies.

## Background

An epidemic is a health emergency that affects the community, not just biologically, but psychologically as well [1]. In the past twenty years, different viral epidemics have shocked the world: SARS in 2003; MERS in 2014, and Ebola in Africa in 2014 [1]. During each of these emergencies, the World Health Organization (WHO) implemented a series of actions to identify the crises’ psychological effects on healthcare workers attributable to the increased workload and organizational changes [2].

Since its outbreak in early 2020, the COVID-19 pandemic has left the healthcare system in a critical situation, with repercussions in the clinical field that have made it difficult to manage the new and unexpected context, and in the organizational field as well, where traditional logic’s distortion is evident [3]. Studies had been conducted in many countries before the COVID-19 pandemic to investigate healthcare workers’ emotional state and their experiences through questionnaires and interviews [4, 5]. In Australia, rapid reviews were conducted on similar topics [6]. In Italy and China, quantitative studies were conducted to analyse healthcare workers’ psychological disorders and the support necessary to improve their well-being in the workplace [7–11].

In addition, a mixed methods study was conducted in the US to assess healthcare workers’ experiences during the COVID-19 pandemic, which collected questionnaires and individual stories [12]. The attention to healthcare workers is related not only to the need to protect their health and avoid infecting their family members, but also to limit the virus’s spread among patients undergoing hospital treatment [13]. If we turn our attention to a specific clinical area, oncology, we find that very few studies have investigated the difficulties that nurses have helping patients with cancer.

Although cancer patients represent one of the most vulnerable populations, and suffer a high mortality rate, a clear management and care approach had not yet been defined following the COVID-19 pandemic’s outbreak [14–16].

National and international scientific societies have developed specific recommendations to prioritise cancer treatment and mitigate the pandemic’s adverse effects on cancer patients’ management [17, 18]. Despite these suggestions, all nurses in the Italian oncological setting faced individual difficulties in caring for cancer patients. Thus, it was critical for them to use resilience strategies and coping mechanisms to overcome the new professional and personal reality and implement project interventions designed to provide emotional, professional, and organizational support [15, 19].

## Methods

### Design, sample, and setting

We conducted a mixed-methods study to evaluate the resilience and coping strategies of nurses working in the oncology setting. Both quantitative and qualitative data were collected and analyzed sequentially, beginning with a quantitative design and progressing to address the phenomena of interest with a qualitative study [20]. The goal of the quantitative study was to highlight the resilience strategies and coping mechanisms that nurses adopted during the pandemic period to cope with stressful events. The qualitative study’s objective was to explore the nurses’ individual experiences in coping with work, organizational, and relational changes following the outbreak of the COVID-19 pandemic.

We used a convenience sampling approach in the quantitative study, with active participation from both a National Cancer Institute (IRCCS Centro di Riferimento Oncologico Aviano - CRO) and a regional hospital situated in northeastern Italy (Azienda Sanitaria Universitaria Friuli Centrale -ASUFC). Subsequently, a purposive sampling strategy was used for the qualitative study. The study’s participants comprised nurses actively employed actively within the clinical oncology context.

### Quantitative data

The study enrolled nurses with the following inclusion criteria: (a) worked during the COVID-19 pandemic, i.e., from March 2020 to March 2021, in the oncology setting (Medical-Surgical Oncology, Oncohematology, and Radiation Therapy) of the National Cancer Institute or the “Santa Maria della Misericordia” general hospital in Udine, northeast Italy; (b) worked with a full-time or part-time employment contract, and (c) gave their informed consent to participate. Nurses who did not meet the criteria above were excluded from the study. All nurses who fulfilled the inclusion criteria were approached through their work e-mail address. Overall, 164 of 269 eligible nurses agreed to participate (participation rate: 61%). According to the study protocol, this sample size was sufficient to achieve the desired precision of the mean resilience and coping scores.

The following self-reported questionnaires were administered to nurses through the Research Electronic Data Capture (REDCap) software [21] to assess their resilience strategies and coping mechanisms:

- The Italian version of the Connor-Davidson Resilience Scale (CD-RISK) [22, 23], a validated tool that includes 25 items measured on a 5-point Likert scale: totally disagree (1); rarely agree (2); somewhat agree (3); quite agree (4), totally agree (5). The scale assessed the way the person felt during the past month (minimum score 25, maximum 100);
- The reduced Italian version of the Coping Orientation to Problems Experienced (COPE-NVI-25) [24] which assessed the individual’s behaviors when nurses experienced stressful or changing events or situations. It includes 25 items scored on a 4-point Likert scale that ranges from (1) I usually don’t do it to (4) I almost always do it. According to the COPE-NVI-25 manual, an overall score was obtained for each subscale and then summed according to identify 5 coping behaviors (minimum score 25, maximum 100).

Further, a questionnaire was used to collect the participants’ background data, including gender, age, hospital unit, years of work experience, and educational qualifications.

### Statistical analysis

The quantitative data were analyzed using SAS (Statistical Analysis System) v. 9.4. The CD-RISK and COPE-NVI-25 scores were standardized according to the respective manuals and expressed on a 0-100 scale, on which 0 indicates the least resilience or ability to cope. The scores were calculated separately for the different subscales. The CD-RISK and COPE-NVI-25 scores were reported as mean values with their corresponding standard deviation (SD); differences across strata were evaluated through the analysis of variance (ANOVA). Linear constraints were used to test the linear trend across strata. Significance was claimed for *P* < 0.05.

### Qualitative data

A phenomenological study was performed until saturation to explore the nurses’ coping experiences [25, 26]. The inclusion and exclusion criteria were the same as those for the quantitative study. Furthermore, participants were chosen based upon their responses in the quantitative study to provide supplementary qualitative elucidations.

Semi-structured interviews were conducted [25, 26] to explore the personal experiences of nurses who cared for patients during the COVID-19 pandemic. A researcher conducted the interviews in a specified room of the hospital, guided by a list of questions the research team developed (Table 1). Examples of questions are as follows: (1) In what area/aspects of care did you spend/commit the most energy? (2) What situations affected you emotionally? (3) What doubts did you have about the decisions you had to make? (4) What did you do to improve? (5) What happened to your personal life with respect to commitments and/or relationships? (6) What happened in your personal life with respect to family and/or friends? (7) Can you think of a metaphor to describe the way that you felt?

Each interview was recorded and transcribed verbatim subsequently. The duration of the interviews ranged from 13 to 57 min (mean: 26 min; SD: 12.0).

Colaizzi’s framework (1978) of seven steps was used for content analysis: (1) three researchers read the text independently to obtain a general overview of the content; (2) meaningful statements were identified; (3) results were evaluated and validated through group discussions; (4) meaningful statements were organised; (4) meaning units were identified; (5) an experienced qualitative researcher assessed the meaning units, sub-themes, and themes; (6) the participants were invited to return and review the interview transcripts and discuss the emerging findings, and (7) general statements were defined to summarize the participants’ lived experiences [27].

**Table 1:** List of questions that guided the interview for the qualitative study

Main questions	Sub questions
1. To begin, you want to introduce the field in which you work or describe your role briefly and whether it changed during the COVID-19 emergency.	
2. In what area/aspects of care did you spend/commit the most energy (practical technical, relational, emotional, educational)?	Give me some examples: What happened in the activities you did every day? What weighed on you the most in the activities you did every day? What went well?
3. What situations affected you emotionally?	Describe, and if possible, give examples
4. What doubts did you have about the decisions you had to make?	Describe, and if possible, give examples
5. What did you do to get better?	Give examples if possible, of the behaviors and activities (strategies) you used to get better. From whom/what did you get help and what did you do?
6. What happened to your personal life regarding commitments and/or relationships?	Could you give some examples? How did you feel … (anxiety, insomnia, other physical or psychological problems)? What have you done to deal with this situation in your life?
7. What happened to your personal life with respect to family and/or friends?	Could you give some examples? How did you feel? What have you done to deal with this situation in your life?
8. Can you think of a metaphor to describe how you felt?	
9. In conclusion, is there anything you would like to add to complement what you have told us?	Is there anything you would like to ask? Is there anything that we have not mentioned, but that is important to you? Thanks again for your time and contribution. At the end we will have a meeting to share the results.

### Ethics

The Ethical Committee of Friuli Venezia Giulia approved the study protocol (June 1st 2021/CEUR-2021-Os-110). Written consent was obtained from all of the participants, and data confidentiality was guaranteed in accordance with Italian rules and regulations, and consistent with the requirements of the Ethical Committee that approved the study. The questionnaires were anonymous. During the interviews, we did not record the nurses’ names, but used a confidential code instead. The study was conducted in compliance with the Declaration of Helsinki principles and Good Clinical Practice.

## Results

### Quantitative data

#### Participants’ characteristics

Overall, 164 nurses agreed to participate (response rate 59.6%); the majority were women (88.4%) and worked for ≥ 30 h/week. The age groups were distributed proportionally as follows: less than 40 years old (32.6%), between 40 and 49 years old (29.2%), and less than 50 years old (38.2%). A combined total of 47% reported professional degrees and were employed within the medical oncology field (43.3%). Refer to Table 2 for a comprehensive presentation of the sociodemographic characteristics of the nurses involved.

**Table 2:** Connor-Davidson resilience scale (CD-RISC) according to demographic and occupational characteristics

Characteristics	*N*	(%)	CD-RISC Mean (SD)	ANOVA
All	164		93.5 (12.9)	
Gender				
Women	145	(88.4)	92.8 (12.8)	*P* = 0.06
Male	19	(11.6)	98.7 (12.6)	
Age (years)				
< 40	47	(32.6)	91.2 (11.4)	*P* = 0.40
40 to 49	42	(29.2)	94.9 (15.2)	
≥ 50	55	(38.2)	93.6 (12.5)	
* Missing*	*20*			
Education				
Professional degree	77	(47.0)	92.3 (12.3)	*P* = 0.04
University degree	56	(34.2)	92.2 (13.9)	
Master/PhD	31	(18.9)	98.7 (11.4)	
Ward				
Medical oncology	71	(43.3)	90.9 (11.7)	*P* = 0.01
Surgery	30	(18.3)	99.3 (11.8)	
Intensive care	13	(7.9)	89.2 (12.2)	
Other	50	(30.5)	94.8 (14.2)	
Length of employment (years)				
< 2	27	(18.1)	91.9 (17.0)	*P* = 0.58
2 to < 10	40	(26.9)	92.2 (11.7)	
10 to < 20	38	(25.5)	95.8 (12.5)	
≥ 20	44	(29.5)	93.4 (12.4)	
* Missing*	*15*			
Working hours (hours/week)				
< 30	12	(7.4)	99.2 (8.7)	*P* = 0.11
≥ 30	151	(92.6)	92.9 (13.1)	
* Missing*	*1*			

### Resilience and coping scores

Nurses reported high resilience (mean CD-RISC score: 93.5, SD: 12.9). The CD-RISK score varied according to education, with a higher mean CD-RISC score for nurses with a master’s or PhD (98.7, SD: 11.4) than those with less education (*P* = 0.04). Similarly, the mean CD-RISK score was higher in nurses working in surgery (99.3, SD: 11.8) than those working in other wards (*P* = 0.01). The difference by gender was borderline significant (*P* = 0.06), and men reported lower CD-RISC scores than women.

The standardized COPE-NIV-25 scores for each sub-scale are reported in Table 3. Transcendent orientation (mean score: 46.8, SD: 27.8) and avoidance strategy (33.3, SD: 12.2) showed the lowest mean score among the subscales considered. Social support and avoidance strategy showed no significant variation across strata. Conversely, problem orientation was higher in nurses aged ≥ 40 years (*P* = 0.01) and in those working in a surgical ward (*P* = 0.05) than in their counterparts. With respect to transcendent orientation, the mean score was higher in women (*P* = 0.03) and in those working < 30 h/week (*P* = 0.03); a significant trend also emerged in the mean COPE-NIV-25 score, which increased with age (*P* = 0.01) and length of employment (*P* = 0.01).

**Table 3:** Coping strategies (COPE-NIV-25) according to demographic and occupational characteristics

	*n*	Standardized COPE-NIV-25, mean (STD)				
		Problem orientation	Social support	Positive attitude	Trascendent orientation	Avoidance strategy
All	164	78.3 (11.8)	70.1 (13.9)	77.4 (12.5)	46.8 (27.8)	33.3 (12.2)
Gender						
Women	145	77.8 (12.1)	69.9 (14.3)	76.6 (12.7)	48.0 (28.0)	33.5 (12.6)
Male	19	82.1 (8.8)	68.5 (9.0)	83.2 (10.3)	32.6 (19.6)	30.0 (9.5)
ANOVA		*P* = 0.17	*P* = 0.70	*P* = 0.046	*P* = 0.03	*P* = 0.29
Age (years)						
< 40	47	74.6 (12.3)	71.1 (11.2)	76.7 (11.6)	38.6 (27.2)	33.5 (11.7)
40 to 49	42	80.1 (10.6)	70.1 (15.2)	76.6 (12.6)	47.8 (28.9)	33.3 (13.1)
≥ 50	55	80.4 (11.7)	69.8 (15.4)	78.0 (13.5)	52.3 (27.4)	32.4 (11.5)
ANOVA^a^		*P* = 0.01	*P* = 0.62	*P* = 0.62	*P* = 0.01	*P* = 0.64
Education						
Professional degree	77	78.4 (11.6)	68.2 (15.0)	76.8 (12.9)	51.6 (27.5)	34.3 (13.1)
University degree	56	77.1 (12.1)	71.2 (11.7)	77.6 (12.5)	38.1 (25.8)	32.7 (11.3)
Master/PhD	31	80.2 (12.0)	73.0 (14.3)	78.6 (11.8)	50.4 (28.9)	32.0 (11.8)
ANOVA^a^		*P* = 0.48	*P* = 0.10	*P* = 0.49	*P* = 0.83	*P* = 0.39
Ward						
Medical oncology	71	76.0 (12.1)	71.8 (13.9)	75.5 (11.7)	47.3 (28.5)	34.1 (11.8)
Surgery	30	83.1 (9.0)	69.1 (14.4)	80.4 (12.8)	52.5 (30.7)	32.2 (11.7)
Intensive care	13	77.4 (9.7)	66.2 (9.2)	73.5 (11.5)	38.1 (21.6)	34.1 (9.7)
Other	50	79.0 (12.8)	69.3 (14.5)	79.4 (13.3)	44.8 (26.0)	32.6 (13.9)
ANOVA		*P* = 0.047	*P* = 0.48	*P* = 0.12	*P* = 0.42	*P* = 0.85
Length of employment (years)						
< 2	27	80.5 (11.9)	70.9 (8.2)	78.0 (13.2)	34.6 (23.6)	33.8 (12.0)
2 to < 10	40	76.2 (11.0)	73.1 (15.3)	75.2 (13.0)	43.2 (28.7)	31.4 (10.0)
10 to < 20	38	78.4 (11.4)	70.0 (14.4)	76.1 (12.5)	52.3 (27.5)	34.9 (13.2)
≥ 20	44	78.9 (13.4)	68.6 (13.5)	78.4 (11.9)	52.6 (26.9)	33.3 (11.9)
ANOVA^a^		*P* = 0.49	*P* = 0.79	*P* = 0.54	*P* = 0.01	*P* = 0.72
Working hours (hours/week)						
< 30	12	83.6 (7.6)	76.1 (14.6)	81.8 (9.9)	63.2 (29.2)	28.1 (11.2)
≥ 30	151	77.9 (12.0)	69.7 (13.8)	77.0 (12.6)	45.1 (27.1)	33.7 (12.3)
ANOVA		*P* = 0.11	*P* = 0.12	*P* = 0.20	*P* = 0.03	*P* = 0.13

^a^*P* for trend

### Qualitative data

A purposeful sample of 15 participants was involved in semi-structured interviews, one man and 14 women aged between 27 and 60 years (Table 4). Content analysis identified five themes that explained the individual experience of cancer nurses’ coping mechanisms: “changing”; “feelings/emotions”; “strategies”; “professionalism and nursing responsibilities”, and “metaphors”. [Place Table 4 here]

**Table 4:** Characteristics of nurses interviewed (*n* = 15)

ID	Age, y	Gender	Education	Department/Hospital
N1	50	Women	RN	Surgical oncology/ IRCCS CRO
N2	43	Women	RN	Medical oncology/ IRCCS CRO
N3	27	Women	BScN	Medical oncology/ ASUFC
N4	38	Women	BScN	Medical oncology/ ASUFC
N5	55	Women	RN	Medical oncology/ IRCCS CRO
N6	54	Male	RN	Medical oncology/ IRCCS CRO
N7	52	Women	RN	Medical oncology/ IRCCS CRO
N8	37	Women	RN	Medical oncology/ IRCCS CRO
N9	29	Women	RN	Radiotherapy/ IRCCS CRO
N10	34	Women	RN	Medical oncology/ IRCCS CRO
N11	53	Women	RN	Triage Service/ IRCCS CRO
N12	26	Women	BScN	Surgical oncology/ IRCCS CRO
N13	54	Women	RN	Medical oncology/ ASUFC
N14	60	Women	RN	Medical oncology/ ASUFC
N15	32	Women	BScN	Surgical oncology/ IRCCS CRO

Abbreviations: ASUFC, Regional hospital -ASUFC; BScN, Bachelor of Science in Nursing; ID, identifier; IRCCS CRO, National Cancer Institute -IRCCS CRO; RN, Registered Nurse

### Theme I: changing

The participants described significant changes in different areas, both work and personal. Organizational changes were necessary to address the spread of COVID-19 and related issues in cancer patients. Triage systems were developed in the cancer centers involved to control patients and visitors’ access to prevent the spread of the virus with the help of other institutions and volunteers. *“[…] we decided to create a kind of “bubble” in the Institute named ‘Triage point’, blocking all access and filtering all those who had to enter the institute and preserve patients and their frailty from the epidemic.” (N11)*.

New outdoor facilities were created to accommodate triage team nurses and physicians, which led to much discomfort for patients and caregivers who had to wait a long while in an unheated and uncomfortable environment. *“We have set up tents and have created the triage station.” (N11)*.

The use of often-deficient Personal Protective Equipment (PPE), the introduction of social distancing, and new procedures for sanitization and hygiene led to several training needs. *“There was difficulty in understanding how we could handle this situation, from a personal protection standpoint, what and how to use PPE, understanding how we needed to approach the patient. “We didn’t know whether to wear the surgical mask or the Filtering Facepiece 2 (FFP2). There was a shortage of masks.” (N8)*.

A new ward and pathway for COVID-19 patients were created, and family members could no longer access hospitals, which led to significant discomfort on the part of both. Further, there were new roles for nurses e.g., triage or swab nurses. Many organizational changes were necessary, such as changes in work shifts, hours, and settings that followed the waves of the pandemic. Nursing staff were taken off the wards and engaged in new activities, such as triage, which put a strain on nurses who remained on the ward and experienced increased fatigue. The presence of novice nurses was also perceived to be a burden because of the increased workload. *“When we had patients with COVID-19 in January, we had to organize the COVID-19 department, which was not planned, so we had to do it based on what they were doing elsewhere at that time and transfer patients.” (N5)*.

New procedures and guidelines were introduced, and the implementation of new practices and rules overwhelmed the business and work organization. Changes were found in work climate and risk perception. *“We had new guidelines that were initially in the draft and then became effective.” (N1)*.

Personal changes involved alteration of biological rhythms, such as sleep-wake rhythm, lack of rest from overwork, and change in daily and family habits. The lockdown forced people to stay at home, which prevented visits to relatives and friends, group or team sports activities, or dancing. Even individual outdoor physical activities were not possible. *“I had disturbed sleep, multiple awakenings […].” (N2)*.

In addition, some nurses experienced isolation from COVID-19 positivity as burdensome, as was the lack of time for themselves. *“The rituals of life were changed. I used to go home and drink coffee at my aunt’s. I eliminated that as well because my aunt was oncology and I thought, ‘I become a danger to my aunt and my parents, I have to stay as healthy as possible, I have to do the shopping for everyone because they can’t leave the house, it’s risky’ […].” (N8)*.

The changes the nurses interviewed reported also affected the relational and communication aspects with the patient. The accounts were discordant. Some nurses experienced difficulties in relationships with patients and their families, and established more fleeting relationships with patients because of lack of time. Others experienced an increased level of communication within the professional team and between nurses and the patient. *“It was a difficult time because people sometimes understood others a little less, the importance of triage and the use of PPE, so you had to explain, always be polite, always be smiling, not always the interlocutor responded appropriately.” (N5)*.

Finally, changes involved increased awareness in seeing the others differently than in the previous period (seeing the colleagues or patients’ needs and weaknesses) in being able to “be there for the other,” in solidarity and mutual help, or in rediscovering that one was stronger. *“I found myself more robust, and that for the way I am, I found it very strange. It was a surprise.” (N15)*.

Additional quotations are provided in Table 5.

**Table 5:** Quotes Theme I: changing

Quotes
“At that time, there was a lot of solidarity because the civil protection departments, the national ‘alpini’ association, the voluntary associations… here at our place, this triage was done very well in my opinion, but it took a huge amount of energy.” (N5)
“We have set up tents and have created the triage station. We created a working team that gave us the strength to face the difficulties of the case altogether; it was not easy to be out in the cold, early in the morning, patients complaining, discomfort, running […] Strategies were found to cope the best we could, we were not prepared, and we had no role models.” We grew into the situation (N11)
“I was immediately asked to train on the proper use of PPE and FFP2 and FFP3 facial filters and also dressing and undressing if one was faced with COVID-positive patients. The use of gowns and socks in accordance with the regional guidelines […] I have noticed an improvement in performing the handwashing procedure.” (N1)
“We structured distinct pathways for COVID-19 patients. Our schedules also changed: we used to finish later.” (N7)
“It was complicated to get patients to follow the rules. They have to stay locked up in their rooms and could not move from the ward, go out because they had to triage again.” (N4)
“The workload increased, and this led to more fatigue. New colleagues were asking for support, and this increased the workload.” (N6)
“The official guidelines changing very quickly.” (N8)
“I had difficulty resting, did not wake up rested, and during sleep dreamed about work.” (N9)
“In the two weeks I was in solitary confinement, yes. It weighed on me to be in the 3 rooms of the house.” (N3)
“Less time for myself.” (N10)
“Colleagues were the only people I could relate to […] With the patient also, because he could not have visitors, he could not have relatives, however, it makes you responsible, it makes you responsible for confidences, and for things that on another occasion he would not have had ways to manifest.” (N15)
“I saw people in a different course [ …] it changed the way of seeing [ …] but also the solidarity that is not such a given thing [ …].” (N7)
“The awareness of the things that helped me to be serene outside of work, to be carefree outside of here.” (N8)

### Theme II: feelings and emotions

The COVID-19 pandemic elicited negative feelings and emotions in nurses. The most frequent was fear of contagion for themselves or their loved ones. Patients had difficulty using the face mask, and maintaining proper distancing put health workers at risk. This behavior had relational repercussions with the patient or their family members, but did not result in hesitation or avoidance. There was also panic among nurses or problems managing the workday, considering the high risk for immune-compromised cancer patients. *“Fear of contagion, especially to family members. In the hospital, we were quite protected, but the fear of home created additional fear and anxiety.” (N2, N6, N14)*; *“We experienced patient discomfort and increased pain, fear.” (N1)*.

Fear also stemmed from the lack of clear guidance on the correct behavior to adopt, the lack of clear and safe procedures and guidelines, and “not knowing” how to work correctly. On the other hand, discomfort increased because of the conflict of values that led practitioners to views either for or against the use of science in emergency management, vaccination, etc. *“A kind of ‘panic,’ in the sense that it was difficult to know how to work as correctly as possible, but because there were no clear and definitive guidelines.” (N7)*.

The nurses reported feeling numerous limitations, such as the obligation to follow orders without having time to check them personally, the inability to manage their own lives through moments of rest, being regarded as machines and not human beings, and the constantly changing knowledge and information. Anxiety increased tremendously because the evolution and consequences of the pandemic were unknown. *“I became frightened by the thought that we are not human beings but machines. Now, after more than a year, we are tired because our lives have changed, and we cannot decide how to handle them.” (N1) “Anxiety and uncertainty due to lack of clear information.” (N4)*.

They also felt the suffering of loved ones and the lack of contact with friends and family. The workload required them to spend a great deal of time at work and they could no longer see friends except on rare occasions. The nurses felt the significant burden of hardship from the hours spent in solitude. *“I was only at home or work; the only people I saw were colleagues from work, and when I got home, I avoided contact. Relationship-wise, it was a big disaster.” (N8)*.

Patients’ death was a powerful experience emotionally. The lack of contact between patients and family members before their death and having communication between the nurses and family members only to return the patient’s belongings was a devastating experience. *“Knowing that some patients didn’t make it was sad… the fact that their belongings were left in the hospital and having to return them to family members… that was the ugliest aspect, the fact that family members hadn’t been able to see them.” (N5)*.

The presence of patients infected with COVID-19 was associated with a very intense and heavy experience for nurses and all healthcare staff. This heaviness was also related to uncertainty and not feeling up to the task or being unable to respond adequately to patients, healthcare staff, and institutions. The fear of not being able to guarantee very high performance and attention during the workday was significant, but having standard procedures to follow made this less difficult. *“I perceived the presence of a much more intense experience. It was also heavy because of the lack of PPE.” (N6); “… with the COVID department, we felt no small burden.” (N10); “… we did not know if we could respond to all the requests and get to the end of the day unharmed.” (N6)*.

Further, the nurses experienced feelings of constant battles over ideological contrasts or values. They also felt fatigued and angry because they were faced with very large numbers of patients to manage during a difficult time. Some nurses regretted not accommodating a patient’s wishes to see loved ones. *“More anger than fatigue: arriving home exhausted and tired […] it was more the anger of this thing, of being in this critical situation at a time of a pandemic, with the very high numbers of patients.” (N10)*.

In contrast, some nurses experienced positive feelings and emotions when they found ways to meet the family members’ needs. *“I am happy because when there was a death, we showed a patient’s body to his family members by taking the body outside the door of the transplant center.” (N3)*.

The excellent work effort during the emergency period created positivity and a feeling of being essential for their contribution or the help they gave to patients or colleagues. Because of this, some said that they felt lucky and had the opportunity to share their tensions with the group, or esteem and trust because of recognition of the services they provided. *“I felt ‘lucky’ because I could do something… I was well. I felt important at that moment because I could contribute.” (N5) “There was a chance to release one’s tension through sharing in the group.” (N6) “[…] the esteem that so many have shown towards me in different situations […] made me aware of the skills I have acquired during this period.” (N8)*.

Supplementary quotations are presented in Table 6.

**Table 6:** Quotes Theme II: feelings and emotions

Quotes
“The fear was of infection. Considering that the spaces were small, there was no opportunity to change the uniform daily. There was doubt: Did I disinfect everything? Did I do everything? This fear brought no hesitation to patient contact while maintaining professional rigor.” (N1)
“There was so much fear of immune-compromised patients; there was more fear for them than for us as staff.” (N3)
“The fear of not implementing the safest procedures. We didn’t know how to deal with this emergency, which enemy we faced. The fear was that PPE would not adequately protect us enough to defend us and bring the virus home.” (N6)
“People were advancing certain ideologies while criticizing those who followed science instead. There was a lack of respect for others; everyone should be able to choose for themselves and not be criticized.” (N1)
“The fact of being left alone.” (N2)
“Anxiety about not knowing how things would turn out. You didn’t know how things would turn out.” (N5)
“It weighed on me not to have relationships with my family members.” (N2)
“In our nursing life, we are always here at work; we see each other with a close friend once or twice a year.” (N6)
“We live day by day. I feel like we are doing everything we can, but it’s not just me, and in confrontation with other people, we continue to battle.” (N5)

### Theme III: strategies

The nurses described certain personal strategies that they used to counteract the suffering that COVID-19 caused in the family, as it was not possible to spend time with friends and relatives. Among them, we found video calls and online shopping. *“Shopping online compensated the impossibility to travel. I start chatting and video calling more. For me it was important the safety of all.” (N1)*.

Dedicating time to activities such as the care of the garden and the house, even if sometimes the energy was lacking and fatigue did not give the opportunity to activate great resilience strategies. *“. I was looking for activities in the house that could occupy free time: garden, vegetable garden… it was not tragic; we have a large house with garden.” (N4)*.

Nurses reported that they had been engaged in activities such as cooking, reading, walking or running, all of which was done to reduce tension. The use of video calling technology also proved effective to communicate with friends and family. *“Maybe I got into cooking more.” (N14)*.

The importance of taking measures to protect family members was also mentioned. Nurses adopted the strategy of distancing even in the home to reduce the possibility of infecting loved ones.

*“First thing I put on all masks and distancing.” (N6) “I have 3 children who did remote teaching, my wife is a nurse and then one goes, one comes, and we had to manage the children who were at home. Parents were close, we saw them in the garden.” (N6)*.

The strategy of limiting contact with friends and family members and remaining alone to minimize the possibility of infection was emphasised. *“I lived 8 months without seeing friends by choice, because I said ‘I work in COVID’, I don’t want to get anyone sick.” (N8)*.

The nurses reported the importance of living and being in the work group as a strategy to reduce the level of tension that also allowed a relational exchange. *“Sitting ten minutes even doing absolutely nothing and exchanging laughter was the strategy I used to reduce them level of tension throughout the day.” (N7)*.

Some nurses reported the innovative contributions they put in place within the working group to help the patient and offer him quality assistance by dedicating time and ensuring continuity of care. *“I think I triggered the mechanism. Then I think I’ve dedicated more time to patients as they rely on you especially when I give continuity to care for several days and then I give continuity to care.” (N3)*.

The nurses reported that over time, it was possible to adopt strategies that countered the situation and the way the possibility of group-building facilitated adaptation. *“Slowly the fear went away and we had to put something in place to counter what happened.” (N3) “The positive side is that the group tried to bring back what was normal before.” (N10)*.

Some nurses adopted proactivity, which is the ability to face and overcome difficult choices, such as the decision to select people in triage to enter the hospital. *“The problem of being sure that everything you do is right is in every decision you make, but you have to take it and go ahead.” (N5)*.

The lack of relatives and caregivers in the wards allowed more agile work for nurses and at the same time, visits were replaced with electronic contacts through video calls. *“That there were no relatives; for the patients it was very sad but we worked more agile. However, telephone contacts were guaranteed, and so we solved the problem and we were happy to have solved it, because our happiness is to satisfy patients in general.” (N6)*.

The nurses reported that often, despite years of work experience, they felt the need to confront each other to ensure safety in the workplace. *“It is part of the assistance to deal with the resources you have at hand; the comparison serves to have safety at work, even if it is 20 years that I work, confront someone makes me go home more peaceful.” (N6).*

Supplementary quotations are shown in Table 7.

**Table 7:** Quotes Theme III: strategies

Quotes
“Let’s say that in this period I was so tired that I didn’t even activate great strategies or resilience, because I was tired.” (N2)
“I remember that I used to take more walks because the tension was so great. When I was in isolation I read more.” (N3)
“The luck of going to work was not little, who was really home for 3 months alone was heavier.” (N6)
“Periodic meetings between coordinators offered the possibility of receiving help without having to ask.” (N7)
“Compared to a patient we showed the mother to, I think I was one of the first nurses to speculate. But if we swab her and let her in, what do you think?” (N3)

### Theme IV: professionalism and nursing responsibilities

Care activities during the pandemic were very complex. They involved all areas, and required a great deal of study to develop new rules and protocols. The nurses played organizational and caregiving roles, with a focus on the relationship with the patient. Much training needed to be conducted, but the most challenging aspect to manage was uncertainty, the need for clear information, fear of being unsafe, and not having up-to-date protocols for reference. However, the nurses’ fear that they or their loved ones would contract COVID-19 did not cause them to hesitate to have contact with their patients while maintaining professional rigor. *“The complexity of care touches all areas, and you have to study new rules and protocols to apply.” (N5, 6, and 10).*

Professional efforts in the organizational area were intense for the triage procedure used to reduce waiting time and meet the patients’ needs. *“I was more engaged in the organizational area, with triage, measuring body temperature, and creating protected pathways for patients, all of which complicated our work.” (N2).*

The relational area was also very challenging for the nurses. The patients were facing difficult situations, and could alternate between moments of anger or despair, which needed to be allayed or supported, and led to difficulties in care management. However, the nurses reported that these moments were opportunities to explain their professional role, network with colleagues or other health professionals, and be recognized with esteem and confidence in their work. They were also times that helped them exchange information or conduct educational meetings. *“I was more involved in the educational activity and in the relational area, as the patients were alone and needed support because of their frailty.” (N1) “If I had time, I tried to be close to the patient.” (N2)*.

Supplementary quotations are presented in Table 8.

**Table 8:** Quotes Theme VI: Professionalism and nursing responsibilities

Quotes
“A few times, I had doubt that I had not worn all the necessary devices to protect myself, perhaps in hectic shifts when there was no time to think.” (N1)
“During my professional activity, my colleagues nor I have ever hesitated to approach a patient for fear of contagion.” (N1)
“We had hard organizational work so that patients did not have high waiting times, so that was the leading work on this for everyone.” (N10)
“The relational part was the most challenging, including explaining the correct mask use.” (N6) “I chatted a lot with the patients. It was almost my need rather than their need. I realized that we could share something that could make us feel good at that moment.” (N15) “From the relational point of view, it was an atomic bomb in the positive sense.” (N8)

### Theme V: metaphors

The nurses described the events they experienced during the COVID-19 pandemic in metaphors, which explained their lived experience clearly.

The image of a journey in which the ship was hit by a furious storm or set adrift represented the shocking event against which one does not give up without a fight. *“I felt like a ship adrift.” (N1) “A storm, something that came and upset us all.” (N3, N11) “But then, the fear disappeared slowly, and we had to put something in place to counter what had happened. So, a quiet storm.” (N3)*.

The nurses emphasized their efforts to keep the ship on course and overcome their fear and fatigue. Further, they began to hope that everything would be over very soon. They expressed the daily toil in responding to patients’ needs and ensuring the effectiveness of health care services.

*“Hopefully, it will end soon […]. I always hoped that everything would end quickly.” (N4) “It will be fine.” (N6)*.

Others used the metaphor of a long winter, a period of darkness filled with hardships and restrictions that gave way to summer when the situation improved at the end of the lockdown.

*“A long winter with little light. Few hours of light in which to focus hope. Head down as when walking in the mountains: head down, and sooner or later, you will get there.” (N2)” I thought about the summer after the lockdown.” (N15)*.

## Discussion

This study integrated both quantitative and qualitative findings on resilience strategies and coping mechanisms that nurses embraced throughout the pandemic period, together with their personal experiences with changes in work dynamics, organizational structures, and interpersonal relationships. The nurses faced many sacrifices and demonstrated remarkable proficiency in managing the challenges that the pandemic engendered. The many changes they experienced served as opportunities to confirm their resilience and coping skills. The array of transformations they encountered provided not only opportunities to exhibit robust resilience and adept coping abilities, but also encompassed instances where they navigated apprehensions about potential infection risks to themselves and their families effectively, thereby ensuring that they provided exemplary care for patients with cancer.

### Resilience strategies and coping mechanisms’ scores

The quantitative study in this research highlighted the resilience strategies and coping mechanisms that nurses adopted to overcome stressful events during the pandemic. The majority of participants were women, which reflects the national trends. In fact, of approximately 460,000 Italian nurses registered with their respective professional orders, 77% are women [28]. The literature does not show a direct influence of gender on the level of resilience, but in this study, male nurses had lower scores. High resilience emerged from the results, significant data for nurses with advanced education, such as master’s or doctoral degrees, which Finstad and colleagues’ (2021) study supported as well [29]. More structured academic courses may have fostered the development of coping and resilience skills or strategies. The literature suggests that high levels of resilience have direct effects on the intention to remain in the firm in which they are employed, life satisfaction, positive affect, perceived social support, adoption of personal precautions against coronavirus, and a mediating role between depression and burnout [29, 30]. The development of emergency and stress coping skills, age, and professional experience were associated with high levels of resilience in this study, and demonstrated also in Croghan et al. (2021) and Di Giuseppe et al.’s (2021) investigations [31, 32]. Primary or continuing nursing education based upon problem-solving methodologies and critical thinking skills may have influenced the results of this study. The influence of age on the development of coping strategies has been demonstrated in the literature [29]. Therefore, younger nurses necessary to increase their skills with appropriate training to improve the management of COVID-19 issues and their coping strategies, which will increase their resilience thereby [33].

### Changes during the COVID-19 pandemic

The qualitative results allowed us to understand the experiences and challenges of nurses engaged in the direct care of cancer patients during the pandemic. The worldwide spread of COVID-19 has influenced their professional and personal lives profoundly [34]. The development of triage systems to monitor access to patients and family members to limit the spread of the virus and the creation of facilities to support them logistically are just some of the organizational changes developed during the pandemic crisis. Further, although guidelines that recommended the use of PPE were put in place, inadequate or limited devices in the workplace have been a major concern [34] because of potential self-infection or transmission of the virus to their family members. New procedures and guidelines were introduced, and their implementation overwhelmed the company and the organization of work. Nurses felt insecure and communication was not always effective [35]. Fear and anxiety related to potential infection increased greatly because the pandemic’s evolution and consequences were unknown. In fact, fear of the new and unknown generated feelings of dread or a psychological state of unrest, particularly in the early phase of the pandemic. This state can elicit emotional symptoms that compromise these people’s mental and physical health, as documented in the literature [36].

New roles were introduced (dedicated triage and swabbing nurses), and their work schedules and workflow were modified. For example, some nurses who worked on the wards were transferred to a facility specifically for virus-positive patients, which left their colleagues short-handed. As found in other studies, the assumption of new roles, increased workload, and responsibilities have created significant discomfort for nurses [37] not only in the field of oncology [36]. Concerns about skills and competencies when nurses are reassigned to different units mandatorily and urgently have been reported and these situations can promote a mix of negative feelings and lead to emotional fatigue and moral distress [38, 39].

An additional change to increase coping and resilience strategies that nurses adopted during the pandemic was related to their private lives. They spent more time tidying the house, tending the garden, walking, and even shopping online. Marshall et al. (2022) confirmed that health professionals have dedicated themselves to gardening, cooking, walking dogs, and taking yoga classes through video to counteract difficulties and reduce stress [34]. Another aspect that emerged was related to the use of video calls with friends, relatives, and caregivers: Some authors have reported that the use of such technology as Face Time or Zoom to reduce the stress attributable to the absence of hospital visits and social gatherings helped reduce the isolation and distance between patients, friends, and family [34, 38–40].

### Quality of care

Despite the perceived excessive workload, nurses reported no reduced quality of care and did not refer to care left incomplete or undone, a frequent problem for COVID_19 patients [41]. In fact, some reported that they developed greater closeness and communication with the patient because of the absence of family members demonstrating high levels of professionalism [42, 43]. However, Hargreaves and colleagues (2022) emphasized nurses’ concern about not being able to provide the same quality of care as before the pandemic [7]. An aspect that did not emerge from our study, but has been reported in the literature [34, 38, 40] concerns the increased use of telemedicine to help patients who could not access hospital care because of COVID-19. The reports emphasised that patients were delighted with the new method of communication. Stockdill et al. (2021) and Zon et al. (2021) also reported the importance of telemedicine during the pandemic [44, 45]. Further, our study highlighted the importance of teamwork to ensure high quality nursing care and facilitate the team’s adaptation to the organizational and work changes the pandemic triggered. Knobf et al. (2022) indicated that nurses demonstrated adaptability, creativity, teamwork, and dedication in caring for patients with cancer [38].

### Strengths and limitations

Only a few multicenter mixed-methods studies in the literature have investigated the resilience strategies and coping mechanisms that nurses adopted during COVID-19. Thus, this study’s contribution in this respect is a strength of the study that can be considered innovative. It represents a significant contribution, as it enhances our comprehension of nurses’ direct experiences throughout the pandemic. The insights drawn from our study carry considerable significance in shaping future practices and directing policy discussions and decisions, all with the overarching goal to provide heightened support to healthcare practitioners during times of crisis.

One limitation is that a self-report survey was used to collect quantitative data, which poses the risk of social desirability bias in the responses, and may have exaggerated the nurses’ tendency to report high resilience. However, self-assessment is considered an essential strategy in multi-method studies. With respect to the qualitative research, some limitations include the voluntary nature of participation in the study and the small number of participants involved, which despite data saturation, may have precluded the possibility of detecting further significant results.

Finally, given that the investigation was undertaken over a year after the epidemiological crisis commenced, the passage of time and the pandemic’s ever-evolving circumstances may have influenced the evolution of the participating nurses’ coping mechanisms and resilience strategies. Consequently, prudent consideration should be exercised when interpreting the findings of this study.

## Conclusion

The COVID-19 pandemic has caused major changes in nurses’ role within hospital contexts: longer work shifts; sudden change of context, and lack of training opportunities to address the emergency situation. In addition, nurses had to cope with the fears and concerns the virus raised, the greatest of which was the fear of infecting their families. However, every professional was able to put in place resilience strategies to counter the situation; group work and discussion with the team proved to be very effective strategies to cope with the emergency and changes and to generate a positive working climate. Few studies in the literature have supported the positive actions of nurses; thus, it would be helpful to implement training to teach professionals coping strategies and resilience and develop clinical research in oncology settings.

## Data Availability

The data sets used and/or analyzed during the current study are available from the corresponding author on reasonable request.

## Abbreviations

ANOVA: Analysis of variance
CD-RISK: Connor-Davidson Resilience Scale
COPE-NVI-25: Coping Orientation to Problems Experienced (25items)
COVID-19: Coronavirus Disease 2019
FFP2: Filtering Facepiece 2
MERS: Middle East Respiratory Syndrome
REDCap: Research Electronic Data Capture
SARS: Severe acute respiratory syndrome
WHO: World Health Organization
